# Primary Perspectives in Meme Utilization as a Digital Driver for Medical Community Engagement and Education Mobilization: Pre-Post Study

**DOI:** 10.2196/40244

**Published:** 2023-01-27

**Authors:** Darrel Wang, Neha Balapal, Amala Ankem, Saishravan Shyamsundar, Adarsh Balaji, Jasmine Kannikal, Marlie Bruno, Shuhan He, Paul Chong

**Affiliations:** 1 Arizona State University Phoenix, AZ United States; 2 CUNY School of Medicine New York, NY United States; 3 Lab of Computer Science Massachusetts General Hospital Boston, MA United States; 4 Geisinger Commonwealth School of Medicine Scranton, PA United States; 5 American University of the Caribbean School of Medicine Cupecoy Netherlands; 6 University of Miami Miller School of Medicine Miami, FL United States; 7 Northeastern University Boston, MA United States; 8 Department of Emergency Medicine Massachusetts General Hospital Boston, MA United States; 9 Center for Innovation in Digital HealthCare Massachusetts General Hospital Boston, MA United States; 10 Campbell University School of Osteopathic Medicine Lillington, NC United States

**Keywords:** buprenorphine, DEA X-waiver, opioid use disorder, OUD, MAT, opioid, waiver, medical education, continuing education, e-training, e-learning, awareness, social media, engagement, marketing, promotion, meme, campaign, advertisement, advertising, outreach, Facebook, Twitter, Instagram

## Abstract

**Background:**

Memes have gone “viral,” gaining increasing prominence as an effective communications strategy based on their unique ability to engage, educate, and mobilize target audiences in a call to action through a cost-efficient and culturally relevant approach. Within the medical community in particular, visual media has evolved as a means to influence clinical knowledge transfer. To this end, the GetWaivered (GW) project has leveraged memes as part of a behavioral economics toolkit to address one of the most critical public health emergencies of our time—the 20-year opioid epidemic. As part of a multidimensional digital awareness campaign to increase Drug Enforcement Administration (DEA)-X waiver course registration, GW investigated the results of meme usage in terms of impressions, website traffic, and ultimately user acquisition, as determined by web-based training enrollment and attendance outcomes.

**Objective:**

The objective of this study was to determine the efficacy of implementing humor-based promotional content versus the traditional educational model, and how the translation of the increase in engagement would increase the participant count and website traffic for GW’s remote DEA-X waiver training.

**Methods:**

The approach to this study was based on 2 time frames (pre- and postcampaign). During April-July 2021, we developed a campaign via advertisements on Facebook, Twitter, Instagram, and the GW website to expand outreach. These memes targeted medical professionals with the ability to prescribe buprenorphine. The time frame of this campaign measured engagement metrics and compared values to preceding months (January-March 2021) for our GetWaivered website and social media pages, which translated to registrants for our remote DEA-X waiver training.

**Results:**

By the end of July 2021, a total of 9598 individuals had visited the GW website. There was an average of 79.3 visitors per day, with the lowest number of daily visitors being 0 and the highest being 575.

**Conclusions:**

The use of memes may provide a medium for social media engagement (likes, comments, and shares) while influencing viewers to pursue a proposed action, such as e-training registration.

## Introduction

### Background

In combating the opioid crisis, buprenorphine is the drug of choice when adhering to an evidence-based clinical standard of care. However, the Drug Addiction Treatment Act of 2000 mandates those clinicians receive a waiver known as the Drug Enforcement Administration (DEA)-X waiver in order to prescribe buprenorphine [1]. Due to the many historical accessibility barriers associated with obtaining a DEA-X waiver, only about 5% of physicians in the United States have acquired this prescriptive authority, which, in turn, limits the ability to care for patients with opioid use disorder (OUD) [2]. GetWaivered (GW) was founded to address this caveat by encouraging and facilitating more clinicians in obtaining their DEA-X waiver. Based upon exploratory research, GW’s model focused on behavioral nudges to address identified barriers as to why clinicians do not obtain their “X” waiver. They include (1) the absence of a social norm, (2) hassle bias in obtaining the waiver, and (3) a lack of salience in treating OUD [3].

Additionally, learning barriers were amplified in the context of the COVID-19 pandemic, as social distancing practices further limited access to traditional medical education, including the DEA-X waiver training courses. Therefore, there has been a push toward web-based platforms for medical education. Outreach efforts since the COVID-19 pandemic have also moved toward social media platforms due to the increase in clinician use [4]. A previous digital campaign performed in April-May 2020 showed that 13% of traffic to the GW website came from social media sources (Facebook, Instagram, and Twitter). Evaluating social media engagement can be key to increasing awareness of our course [5].

Twitter, a social media platform, has around 340 million active users, with about 500 million Tweets (microblogs) posted per day in a wide variety of locations and languages [6]. Twitter is one of the most popular forms of social media used for health care communication, which is why more people are continuously using it as a data source for research [7]. Twitter’s ease of rapidly distributing published information, accelerating peer review, and engaging large numbers of people from various communities make it a key player in professional education in health care [7]. Despite the constant evolution of social media platforms, advertising, and marketing, humor has been a staple that has withstood the tests of social media evolution. The engagement that is provoked when humor is at the forefront of any message allows for reader engagement through collective laughter and relatability [8]. A meme can be best described as a photo with a witty caption. Memes are often created to be pop-culturally themed pictures with words that are either captioned or overlaid on the photo. GW was able to target memes created with an emphasis on modern-day findings of the behavioral aspects surrounding the viewership of the artwork via focal viewpoints [9]. The campaign focused on the effectiveness of a text overlay on top of a photo that is widely known in popular culture. The focal viewpoint, in the case of meme content creation, shifts the focus from examining a picture to simultaneously reading an in-figure caption. The pop-culture references, which entailed largely popular pictures, were overlaid with words that provided comical references and statements to encourage reaction formation from audience members. Measures included engagement from the health care community via social media.

Social media is both collaborative and user-generative focused, which promotes participatory learning and action among its users [10]. Existing public engagement with scientific social media is one-directional, but increasing participation through creative posts and discussions can provide new avenues of communicating with target audiences [11]. However, quantifying the impact of social media on health campaigns has proven to be a challenge [12-14]. Overall, web analytics can contribute to determining a website’s usability and conversion rates [15]. Specifically, for evaluating the efficiency of Facebook campaigns for large-scale public health recruitment, such as for COVID-19, cost per click and cost per response are effective outcome measures [16]. Furthermore, while the measurement approaches for measuring social media can be quantitative, qualitative, or mixed methods, it is recommended that the value of social media in a health care endeavor be evaluated by analyzing pre- and post-social media adoption [17].

Data surrounding the efficacy of memes as the content of an outreach campaign have been lacking, so we sought to analyze data on the use of memes to engage social media users in a mission to drive DEA-X waiver registration and, in turn, increase the number of nationwide clinicians that have fulfilled mandated requirements to prescribe buprenorphine. Overall, this study is important for both theory and practice [17], as it contributes to the accumulation of knowledge in the emerging field of health care social media analytics while also enabling the GW team to design social media strategies that yield enrollment results.

### Objectives

Our first aim was to create a meme-based social media approach to reach greater audiences while using a digital framework methodology for increases in viewer count and the translated registration numbers for our e-training [18]. This first step resulted in the creation of memes by our content creation team. Additionally, we were able to assess the study’s time frame for participants based on their previous registration numbers. We examined if there were objectively better reaches to our audience and assessed the sustainability and efficacy of the process.

Our second aim was to analyze if the methodology could create more engagement from the community in the form of post likes, shares, and training registrations. The laugh model was used (Table 1) as a method of community outreach that resulted in relatability to pop-culture references to drive social media engagements [19]. The objective of the laugh model was to implement a low-cost method of promotion for public health crises. The framework primarily focused on the communication of public health issues through humor and pop-culture references. The model itself sought to increase awareness, as highlighted on [20]. The conclusions from this study indicate an increase in effectiveness when humor-based promotional measures are compared against education-focused efforts. The laugh model’s part in GW’s desire to increase participation in DEA-X waiver training would allow for the empowerment of humor-driven marketing tactics to increase viewership. The method, in turn, would increase participation in GW’s web-based training for understanding the DEA-X waiver logistics and buprenorphine mechanisms.

**Table 1:** Use of the laugh model to promote GetWaivered awareness.

Framework component	GetWaivered campaign
Consumer motives	Need for more DEA^a^-X waivered clinicians to help treat opioid use disorder to help curb the opioid epidemic Need to spread awareness on an easy-to-share web-based platform with engaging content
Priority health needs	Eligible clinicians need to obtain a DEA-X waiver in order to prescribe buprenorphine, an evidence-based treatment for opioid addiction. These clinicians need to be nudged and made aware of the need to become waivered
Message development	Use of memes and other shareable content to encourage people to visit the GetWaivered website
Web 2.0	Use of Facebook, Twitter, and Instagram platforms; Twitter outperforms the other platforms in reach and engagement and has a larger following.
Social momentum	Increased content engagement results in a sharing, retweeting, and organic user-generated advertising “domino effect,” with viral content inducing substantial surges in social media traffic.
Public health impact	Total website visitors (April 1, 2021-July 31, 2021): 9598 E-training registrants (May 2021-July 2021): 396
Sustainability	GetWaivered meme creation and implementation expense: US $600 per month, with ongoing social media account maintenance performed voluntarily in-house at no cost.

^a^DEA: Drug Enforcement Administration.

## Methods

### Ethics Approval

The study was approved by the Mass General Brigham review board (#2021P000447). The procedures followed were in accordance with the ethical standards of the responsible committee on human experimentation (institutional and national) and with the Helsinki Declaration of 1975, as revised in 2000 (5).

### Target Audience

The intended target of the social media postings was clinicians who can prescribe buprenorphine. This included attending physicians, resident physicians, nurse practitioners (NPs), physician assistants (PAs), and health professional school students [21]. These students (NP, PA, and medical) and residents, upon completion of their degree plans, will receive their DEA-X waiver upon the completion of their training, as well as their official license numbers. The evaluated Twitter, Instagram, and Facebook posts were posted between April 2021 and July 2021 and analyzed by metrics including engagement, users, acquisition, content, and platform [15].

### Social Media Program, Content, and Promotion

Research supports the incorporation of social media as a viable channel capable of integrating cohesive messaging and furthering mission-driven organizational objectives, particularly as they relate to public health and welfare [12]. GW sought to leverage these insights to empower clinicians in treating OUD through evidence-based interventions via a multidimensional model based on capacity-building.

#### Project Design

GW first sought to expand outreach efforts through its social media interface before using memes to advocate for DEA-X waiver course enrollment. This biphasic model consisted of 3 primary digital activities: (1) conceptualization of organic content with a unified call-to-action, (2) deployment of this material through a series of timed, course-specific social media advertisements to motivate follow-through, and (3) real-time monitoring of course registration through the course registration page [22] to inform strategic responsiveness in terms of creative direction and distribution methodology. The first stage of the model focused on the generation of memes, based on pop-culture references, and the dissemination of information via social media. The second stage of the model focused on the comparison of numerical values of social media and website engagers, to previously measured numbers prior to humor-based promotions. The target audience was clinicians with current or prospective eligibility to prescribe buprenorphine. This included attending and resident physicians, NPs, and PAs, as well as students in each of these respective cohorts [21]. Residents and students were incentivized to complete GW’s waiver course preemptively to streamline the waiver acquisition process upon degree completion and conferral of licensure. The variables of interest, including but not limited to engagement, user acquisition, content-associated metrics, and overall platform data, were measured during the 4-month period from April 2021 to July 2021 from GW’s Twitter, Instagram, and Facebook accounts [15].

#### Social Media Programming: Content Conceptualization, Delivery, and Promotion

**Figure 1:**
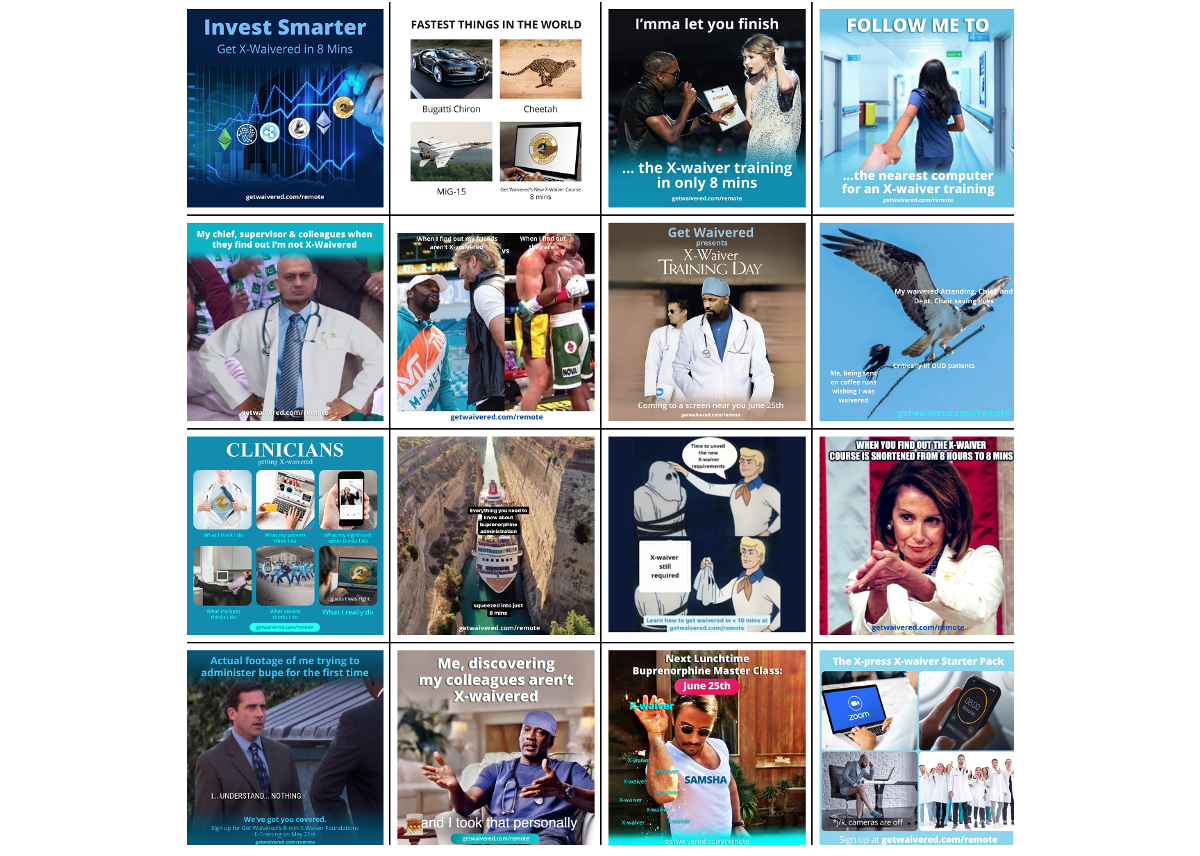
Examples of memes.

Based on the laugh model (Table 1), humor-embedded informational content was presented to an audience segmented according to prevalent attitudes toward buprenorphine use in the treatment of OUD. This included those who (1) were unaware of the intervention, (2) were in favor but unmotivated to get DEA-X waivered, (3) were undecided about its adoption, and (4) expressed opposition. For those in category 1, the main goal of message development was to educate; this consisted of presenting objective information about buprenorphine, its administration, and its proven clinical efficacy. Regarding those in category 2, an emphasis was placed on the support GW provides to minimize the hassle and bias associated with obtaining the waiver. Appealing to those in category 3 involved persuasive tactics directed toward altruistic drivers of behavior and a sense of solidarity with peers and role models within the medical community. Lastly, engaging those in category 4 depended on rebuttals that challenged common arguments through myth-busting graphics and captions. To maximize target audience internalization, time-relevant memes (Figure 1) were leveraged as a vehicle for this messaging, to exploit viral trends and influence perception as less confrontational or argumentative.

**Figure 2:**
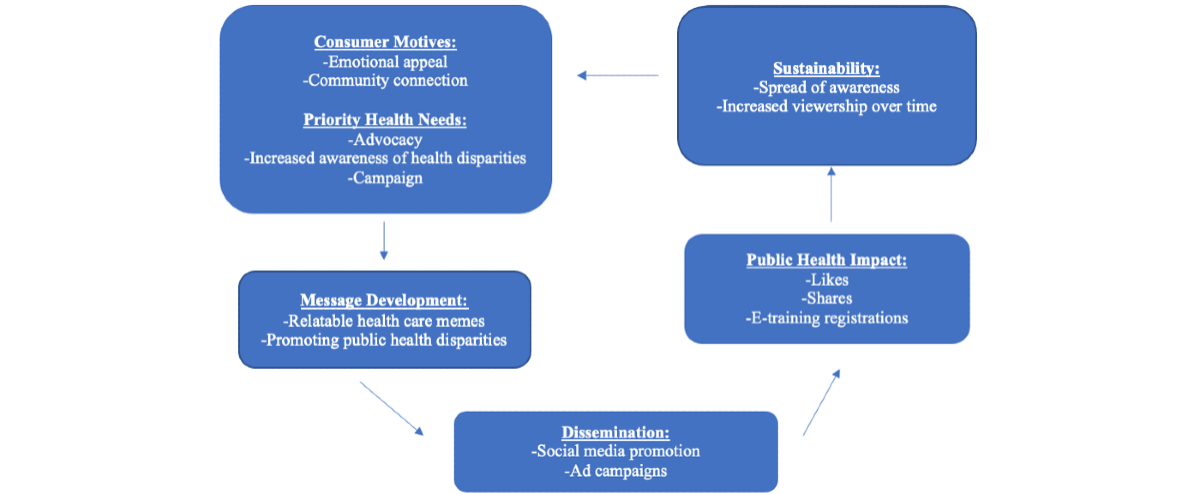
Meme dissemination framework.

GW’s meme content, in addition to flyers, was delivered in regularly spaced intervals to avoid overwhelming followers with a flux of posts all at once, or a lack thereof, resulting in user attrition. The goal of this social media schedule was 2-fold: to increase the number of followers through a consistent digital presence and promote GW website traffic to capitalize on another opportunity to influence primed behaviors (eg, waiver course enrollment). Although the decision-making stage of the social media user affects the likelihood of an endorsed action, consecutive reinforcement via multiple digital touch points can exponentially increase this probability (Figure 2).

Content promotion on Facebook, Instagram, and Twitter was executed through both direct and indirect strategies. The direct promotion was achieved primarily through advertisement investments on Facebook and Instagram. The content was “boosted” among a custom-selected audience that varied by geographical location, clinical position, interests, and demographic variables. Furthermore, the promotional campaign duration was defined according to the days between postlaunch and the next e-course date to maximize enrollment.

Moreover, it is important to note that these efforts were not mutually exclusive; due to the integration of social media, GW-branded content was cross-promoted across platforms simultaneously. For instance, in the process of making a post “live” on Instagram, the option to share it on Facebook was also selected; Facebook users are alerted to the Instagram account source when this content appears on their respective feeds, which has the potential to convert the Facebook user into an Instagram follower as well, as a consequence of a single content consumption experience.

Indirect methodologies depended on a network of recruited influencers within the health care space, further segmented by professional status (eg, a medical student), professional specialty (eg, an NP), or organization (eg, the Florida Medical Association). In addition to tagging high-reach influencers directly on an image, they were also sent direct messages with a gentle request to repost either the image itself, an e-flyer, or an active story presenting the same image or training-related information. Hashtags were also leveraged as a means to increase exposure to GW’s target audience.

#### Mobilization

The success of our call to action through social means was evaluated based on both registration and participation in our DEA-X waiver training e-course. Of those in attendance, pharmacology and administration were taught through instructional and clinical case-based learning pedagogies by certified experts in addiction medicine. Course instructors then prepared the graduates for the next phase of mobilization by providing a roadmap on how to navigate the administrative aspects of obtaining a DEA-X waiver. The first step, submitting a Notice of Intent form, was done in class, enabling any issues to be addressed instantaneously. Prompting clinicians to initiate the process in class made them more likely to follow through to completion. The training concluded with both local and national resources to support clinicians with any concerns or questions, in addition to a post-course email and our social media linking back to this same information so that it was readily accessible through multiple interfaces.

The flowchart illustrates the self-servicing, repetitive cycle that the promotional meme content is based upon. The success of the cyclical nature of the process is dependent on consumer engagement, which results in course registration for clinicians.

The compilation of memes provides several examples of successful engagement outreach from the GW social media avenues (Twitter, Instagram, and Facebook). With trending pop-culture photos, GW was able to tie visual representations of emotions and actions to the relatable text regarding the efficacy of GW’s web-based DEA-X waiver training.

The pie charts illustrate the demographic breakdown by self-identified gender and the differences between the populations of returning web visitors and first-time web visitors.

### Data Collection and Analysis

The assessment of website metrics, including page traffic and course registration, was gathered, and each impression was noted. These measures allowed for a general understanding of the impact that each promotional post had on training registration. In addition, the timelines associated with meme launches coincide with an improvement in outreach, as seen by the analytical trends.

Users are defined as visitors who have started at least one session with a website during a specified period. A session is defined as a group of user interactions with a website. Page views, also known as page impressions, are defined as the total number of views a website has had. Google Analytics defines direct traffic as website visits that arrived on your site either by typing your website URL into a browser or through browser bookmarks. Organic search traffic is defined as website visits resulting from unpaid listings in search engine results. Social traffic is website visits through social media networks. Referral traffic is defined as website visits through other website domains. Google Analytics uses “unique identifiers” through the use of cookies to associate website visits with a particular user [23]. Google Analytics data accessible through the GW account were used to analyze GW website traffic data.

Data were compiled to assess the level of outreach. By monitoring both viewership and engagement, 2 layers of understanding could be gathered. The first aim was to determine the success of views among the target demographic. The second aim focused on how many of those users found the content interesting enough to engage or sign up for. By analyzing both of these variables, points of improvement were identified and adjusted to understand the full potential of humor-based advertising.

Based on the successful increase in social media engagement, which subsequently resulted in more frequent website traffic, according to the laugh model [19]*,* an analysis of visitors to the GetWaivered website was compared to previous data before the implementation of humor-based advertisements was explored. The data was compiled to analyze daily visitors and the number of views per post. The demographics were further broken down into return users and new users. By assessing the chronology of posts and viewer retention, the team was able to assess the trends and growth of outreach at the end of the research period.

## Results

From April 2021 to July 2021, there were a total of 9598 visits to the GetWaivered website. There was an average of 79.3 visitors per day, with the lowest number of daily visitors being 0 and the highest being 575 (Textbox 1).

The website page that was visited the most was the course registration page [22], which resulted in 5100 views and was where the e-training registration form, dates, and instructions were located.

Table 2 shows the differences between user engagement metrics (page views, users, and sessions) for the GW website in the months preceding the meme campaign (January to March 2021) and the meme campaign (April to July 2021), in addition to their respective *P* values. During the campaign dated April 2021 through July 2021, the statistical analysis has reflected an increase of 7602 in page views (*P*=.009), an increase of 5878 new users (*P*=.002), and an increase in website sessions of 5990 (*P*=.003).

**Figure 3:**
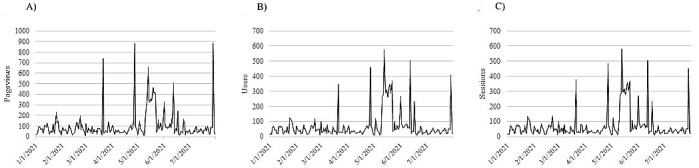
GetWaivered pageviews, users, and sessions from January 2021 through July 2021. A timewise display of user engagement metrics (A: pageviews; B: users; C: sessions) shows substantial increases in metrics from January 2021 to March 2021 versus April 2021 to July 2021.

**Figure 4:**
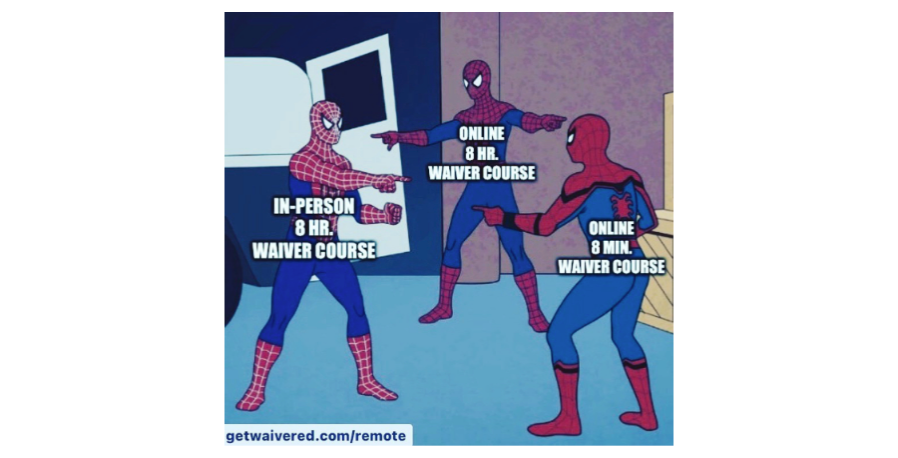
Memes correlated with GW’s largest e-course. GW: GetWaivered.

The page received its most daily website traffic (575, as previously noted) on May 21, 2021, after the social media post, as indicated in Figure 3, was disseminated. After the creation and dissemination of Figure 4 and the website receiving its most daily visitors, the month of May 2021 resulted in our greatest number of e-training registrants (243).

**Website traffic analytics.**

**Website visits from April 2021 to July 2021**

- Total visits: 9598
- Daily average: 79.3
- Daily high: 575
- Daily low: 0

**Table 2:** Timewise comparison of GetWaivered user engagement metrics.

	Page views	Users	Sessions
January-March 2021, n	6853	4287	4562
April-July 2021, n	14,455	10,165	10,552
Difference (*P* value)	7602 (.009)	5878 (.002)	5990 (.003)

## Discussion

### Principal Findings

Our findings are key to informing future GW social media strategies and also provide an outline for other public health social media accounts, especially those targeting medical education. Since methods for analyzing social media use in public health are varied, these preliminary findings provide a basis for developing social media posting structures and evaluating them for future projects.

Website analytics were gathered prior to the July 22, 2021, e-training to assess the website traffic. The discontinuation of the data analytics was due to the removal of promotional means (eg, memes) before the scheduled July training and allowed for analytics and comparisons of training, both with and without promotion. Data on other population indicators, such as gender, age group, and location of followers on social media, were also gathered but not specifically analyzed during the time of this study.

The target audience was able to comment, react, and especially share information after viewing the memes. The opportunity for the viewer to share the post would provide a wider network of people to have access to the training.

Another important feature of our study was that the targeted population had the opportunity to register for training immediately after viewing the memes (Figure 4). This option for immediate action by providing the registration link in the description of the posts would remove any questions about what his or her next steps may look like. The individual was not only allowed to share the information but to also take a personal step in his or her career to incite change in the local community.

### Conclusions

The incorporation of the laugh model framework assisted in the promotion of GW’s remote digital DEA-X waiver training. The correlation between our pre- and postmeme promotional metrics showed the potential for our meme-based strategy as a method for community health intervention.

This finding shows the potential for humor-based promotional methods to show effectiveness in community engagement when compared to previous methods of educationally based promotions. This adds to the evidence that digitally native approaches can be a large driver in the future of promotional approaches that GW will use to enhance and increase course registrations and may be applied to other scenarios where similar communication is required.

### Limitations

One limitation of this study is that the data used for analysis is for 121 days. Future studies will focus on specific meme campaigns that span longer periods and throughout more GW courses. Furthermore, we have not yet deployed website registration tracking from the links in the social media posts, but we were able to track total course registrants in real-time. However, we aim to have the tracking implemented in future courses to be able to add clarity to the relationship between social media memes and actual course awareness. Memes and comedic posts are only one form of engaging social media content. There are a variety of other engaging posts that can be used, such as GIFs, article retweets, incentive advertisements, positive messages or stories, and influencer posts. We suggest further research to investigate these other avenues and compare memes to other forms of engaging posts as well.

### Comparison With Prior Work

Social media can and has been a useful tool for multiple public health efforts, as it allows not only for users to engage and interact with one another on social media platforms but can also allow for marketers, such as public health organizations, to engage with the users as well [24]. Public health organizations have been steadily improving their digital presence, but several of these social media platforms are not impactful due to a lack of user engagement [19]. Various studies have been done on the different uses of social media by using both memes and social media influencers to impact change. One study done in relation to the Truth Initiative measured the interactions from tweets and memes by social media influencers to discourage the use of tobacco products [25]. Memes, such as “CATmeggon,” were made to have a positive and comedic tone and were found to reach close to 1.5 million people per day [25].

As social media users are more likely to use social media platforms as a means for passing their time or simply as means for instant gratification, one study recommends that public health organizations use the laugh model in order to engage with users [19]. The laugh model that was recommended in the study incorporates factors such as humor, viral content, and entertainment to effectively promote information to the public [19]. Social media campaigns have been used to increase awareness of the COVID-19 pandemic [12]. One study reported that social media was an essential tool in promoting behavioral changes and ways to increase protection against the novel virus [12]. Various studies have been done on the different uses of social media, using both memes and social media influencers to impact change [25]. One study done in relation to the Truth Initiative measured the interactions from tweets and memes by social media influencers to discourage the use of tobacco products [25]. Another study by Brown et al [26] incorporated memes into pharmacy education. The study revealed that the incorporation of memes, though impactful in engaging with the current-age audience, needs more research to be done to make any conclusions on their effectiveness [26]. Our study shows a substantial increase in the number of clinicians who were able to attend the DEA-X waiver training by simply framing public health issues using the methods discussed above.

### Future Work

The future work of GW focuses on expanding social media campaigns and outreach to increase viewership and engagement. Our GW team plans to deploy more engaging content posts and memes and increase promotional spending on Facebook to reach more people. We can also focus on action plans for long-term engagement with users and collecting data from future courses for prospective studies. Furthermore, we aim to investigate the ideal combination of social media platform strategies for course enrollment and the impact of additional social media platforms such as LinkedIn and YouTube. This evaluation provides preliminary data and a framework as a baseline for planned future studies.

## Abbreviations

DEA: Drug Enforcement Administration
FORE: Foundation for Opioid Response Efforts
GW: GetWaivered
NP: nurse practitioner
OUD: opioid use disorder
PA: physician assistant
